# Factors Secreted by Endothelial Progenitor Cells Enhance Neurorepair Responses after Cerebral Ischemia in Mice

**DOI:** 10.1371/journal.pone.0073244

**Published:** 2013-09-04

**Authors:** Anna Rosell, Anna Morancho, Miriam Navarro-Sobrino, Elena Martínez-Saez, Mar Hernández-Guillamon, Silvia Lope-Piedrafita, Verónica Barceló, Francesc Borrás, Anna Penalba, Lidia García-Bonilla, Joan Montaner

**Affiliations:** 1 Neurovascular Research Laboratory, Department of Neurology and Department of Internal Medicine, Universitat Autònoma de Barcelona, Vall d’Hebron Research Institute, Barcelona, Catalonia, Spain; 2 Neuropathology Unit, Department of Pathology, Hospital Vall d’Hebron, Barcelona, Catalonia, Spain; 3 Servei RMN, Universitat Autònoma de Barcelona, Cerdanyola del Vallès, Catalonia, Spain; 4 Centro de Investigación Biomédica en Red en Bioingeniería, Biomateriales y Nanomedicina (CIBER-BBN), Cerdanyola del Vallès, Catalonia, Spain; School of Pharmacy, Texas Tech University HSC, United States of America

## Abstract

Cell therapy with endothelial progenitor cells (EPCs) has emerged as a promising strategy to regenerate the brain after stroke. Here, we aimed to investigate if treatment with EPCs or their secreted factors could potentiate angiogenesis and neurogenesis after permanent focal cerebral ischemia in a mouse model of ischemic stroke. BALB/C male mice were subjected to distal occlusion of the middle cerebral artery, and EPCs, cell-free conditioned media (CM) obtained from EPCs, or vehicle media were administered one day after ischemia. Magnetic resonance imaging (MRI) was performed at baseline to confirm that the lesions were similar between groups. Immunohistochemical and histological evaluation of the brain was performed to evaluate angio-neurogenesis and neurological outcome at two weeks. CM contained growth factors, such as VEGF, FGF-b and PDGF-bb. A significant increase in capillary density was noted in the peri-infarct areas of EPC- and CM-treated animals. Bielschowsky’s staining revealed a significant increase in axonal rewiring in EPC-treated animals compared with shams, but not in CM-treated mice, in close proximity with DCX-positive migrating neuroblasts. At the functional level, post-ischemia forelimb strength was significantly improved in animals receiving EPCs or CM, but not in those receiving vehicle media. In conclusion, we demonstrate for the first time that the administration of EPC-secreted factors could become a safe and effective cell-free option to be considered in future therapeutic strategies for stroke.

## Introduction

Stroke remains a major cause of death and disability worldwide. However, the only approved treatments are pharmacological and mechanical reperfusion therapies, which aim to restore blood flow in hyperacute ischemic patients [1]. Although thrombolysis with tissue plasminogen activator is effective and lifesaving, only 2–5% of all stroke patients receive this treatment. Therefore, it is necessary to develop new stroke therapies that could be used to treat a large number of patients in the delayed phases of this devastating disease. In this regard, the idea that neurovascular plasticity could contribute to brain recovery has emerged as a powerful new concept for stroke therapy [2]. Thus, for brain repair after stroke, both angiogenesis and neurogenesis will have to be potentiated in the ischemic brain, and endothelial cells have been established as critical components of the neural stem cell niche. Indeed, they secrete soluble factors that maintain the neurogenic potential of the central nervous system [3] and endogenous angiogenesis has been causally linked to endogenous neurogenesis after stroke in mice [4].

Classically, the formation of new blood vessels was thought to be mediated exclusively by embryogenic vasculogenesis, followed by the sprouting of endothelial cells from preexisting vessels during angiogenesis [5]. However, this dogma was called into question upon the discovery of bone marrow-derived CD34+ cells with endothelial characteristics and circulating in adult human blood [6]. These cells, referred to as endothelial progenitor cells (EPCs), were capable of differentiating *ex vivo* into endothelial-phenotyped cells, and represent a new model for endothelial generation and vessel repair. Thereafter, multiple studies using both *in vitro* and *in vivo* models of angiogenesis have confirmed the role of these EPCs as an alternative cell-based approach to enhance angio- and vasculogenic responses. However, few studies have investigated their role in animal models of cerebral ischemia. In this regard, neovascularization and neuronal regeneration could be successfully induced after delayed systemic administration of human cord blood-derived CD34+ (containing EPCs), in a mouse model of cerebral ischemia [7]. Another study showed that the hyperacute transarterial administration of bone marrow-derived EPCs expanded *ex vivo* reduced both infarct volume and neurological deficit in a focal ischemia-reperfusion rat model, by attenuating endothelial dysfunction [8]. More recently, it was shown that the administration of endothelial colony-forming cells (or outgrowth EPCs) one day after transient cerebral ischemia improved functional recovery by reducing the number of apoptotic cells and increasing brain angiogenesis in rats [9]. Finally, other authors have suggested that the hyperacute administration of EPCs protects the brain against ischemic injury and promotes neurovascular repair, thus improving long-term neurobehavioral outcome through SDF-1-mediated signaling pathways [10]. However, when translated to clinical practice, cell-based therapies may lead to adverse side effects, such as microemboli or tumor formation, lung dysfunction, or abnormal immune system reactions [11], [12]. In this context, it has been proposed that cell-free, but cell-based, strategies could open new avenues in the field of regenerative medicine, and should be explored for stroke treatment [13].

To the best of our knowledge, the potential therapeutic benefits of EPC-secreted factors as cell-free therapies have not been investigated in preclinical models of stroke. Our hypothesis is that a cell-free treatment based on the administration of paracrine factors secreted by EPCs could enhance neurorepair after cerebral ischemia. Therefore, the aim of this study was to demonstrate for the first time that angiogenic treatment with EPCs, or conditioned media (CM) containing EPC-secreted factors, could potentiate cerebral angiogenesis and improve neurorepair after stroke.

## Materials and Methods

### Ethics Statement

The Ethics Committee of Animal Experimentation (CCEA) of Vall d’Hebron Research Institute approved the study protocol (No. 4629), and all experiments were conducted in accordance with the Spanish legislation and the Directives of the European Union. Metamizol magnesium (400 mg/kg) was administered subcutaneously after the procedure to prevent surgery-related pain. The ARRIVE guidelines were considered when designing and reporting the results of the study.

### Endothelial Progenitor Cell Culture

Previously characterized EPCs were obtained as described elsewhere [14]. Briefly, a pool of spleens obtained from BALB/C mice males (Charles River Laboratories, Spain) was mechanically minced, placed at 37°C for 15 minutes in 1 mM EDTA and run through a 40-mm nylon membrane to obtain a cell suspension. Mononuclear cells (MNCs) were obtained by density gradient centrifugation with Ficoll-Paque Plus (GE Healthcare, Sweden), shortly washed with a red blood cells lysis solution (150 mmol/L NH_4_Cl, 10 mmol/L NaHCO_3_ and 0.1 mmol/L EDTA in distilled water) and gently washed with complete endothelial growth medium-2 (EGM-2; Lonza, USA). This media is composed of endothelial cell basal medium (EBM) containing 10% fetal bovine serum, human endothelial growth factor (hEGF), vascular endothelial growth factor (VEGF), human basic fibroblast growth factor (hFGF-b), insulin like growth factor 1 (R3-IGF-1), GA-1000 (gentamicin and amphotericin-B), heparin, hydrocortisone and ascorbic acid. Isolated MNCs were finally resuspended in EGM-2 and 5×10^7^ MNCs were seeded on fibronectin-coated flasks (25 cm^2^) and incubated in a 5% CO_2_ atmosphere at 37°C. Under daily observation, first media change was performed two days after plating and, thereafter, media was changed every 2–3 days.

### Permanent Focal Cerebral Ischemia

Adult BALB/C mice males weighing 25–30 g (Charles River Laboratories) were given free access to food and water prior to surgery. A reproducible model of stroke by middle cerebral artery occlusion (MCAO) affecting the cortex was induced by electrocauterization of the distal portion of the left MCA, as previously described [15]. Metamizol magnesium (400 mg/kg) was administered subcutaneously right after the procedure as analgesic. The duration of anesthesia in all animals was less than 30 minutes. Mice that showed a decrease in cerebral blood flow >75% after the procedure as compared to baseline values were used for experiments. Sham surgery consisted of the same procedure, except that the artery was not electrocoagulated. A total of 44 mice were initially included in the study (40 MCAO and 4 shams). The animals were euthanized at two weeks.

### In Vivo Magnetic Resonance Imaging (MRI)

MRI was performed at seven Tesla in a horizontal magnet system (BioSpec 70/30 USR, Bruker BioSpin, Ettlingen, Germany) equipped with actively shielded gradients capable of 400 mT/m (B-GA12 gradient coil inserted into a B-GA20S gradient system) and a dedicated mouse brain quadrature surface coil, actively decoupled from a transmit volume coil with 72-mm inner diameter. The animals were positioned on a scanner bed, which allowed for a localized delivery of anesthesia (isoflurane, 0.5–1.5% in O_2_ at 0.8 L/min; respiratory frequency was monitored with a pressure probe and maintained at 50–80 breaths/min). A water recirculating system integrated into the animal bed was used to control the body temperature, which was measured with a rectal probe (37±1°C).

T2-weighted fast spin-echo images (T2WIs) were initially obtained in axial, sagittal and coronal planes to be used as reference scout images for reproducible slice selection at each MRI session. Imaging parameters for these images were: echo time (TE) = 12 ms, echo train length (ETL) = 8, effective echo time (TEeff) = 36 ms, repetition time (TR) = 4 s, field of view (FOV) = 1.92×1.92 cm^2^, matrix size (MTX) = 128×128, and slice thickness (ST) = 1 mm. Afterward, coronal MRI sections were acquired over an 8.7 mm block starting 3.2 mm anterior to the bregma and towards the cerebellum. T2WIs were acquired using a fast spin-echo sequence with ETL = 8, TEeff/TR = 60 ms/4.2 s, FOV = 1.92×1.92 cm^2^, MTX = 256×256, 16 contiguous slices with ST = 0.5 mm and a 0.05 mm gap between them. T2 maps were also obtained using a multi-slice multi-echo sequence with 18 TE values ranging from 10 to 180 ms, TR = 4 s, MTX = 128×128, FOV = 1.92×1.92 cm^2^, and slices covering exactly the same brain region as in high resolution T2W coronal images, but with eight continuous 1-mm slices and 0.1 mm gaps. Diffusion tensor imaging (DTI) was carried out using a spin echo 4-shot echo-planar readout sequence with identical geometry as in T2 mapping. Diffusion weighted images were acquired along 20 diffusion directions and 4 b values (b = 0, 200, 600, 1000 s/mm^2^). The imaging parameters were: TE/TR = 35 ms/4 s, diffusion gradient duration (γ) = 4 ms, diffusion gradient separation (γ) = 20 ms, MTX = 64×64, FOV = 1.92×1.92 cm^2^. Maps of the trace of the diffusion tensor, also referred to as apparent diffusion coefficient (ADC), and fractional anisotropy (FA) were derived using Paravision v4.0 (Bruker BioSpin, Ettlingen, Germany) as described previously [16]. The total preparation and scan time for each animal was approximately of one hour.

**Figure 1:**
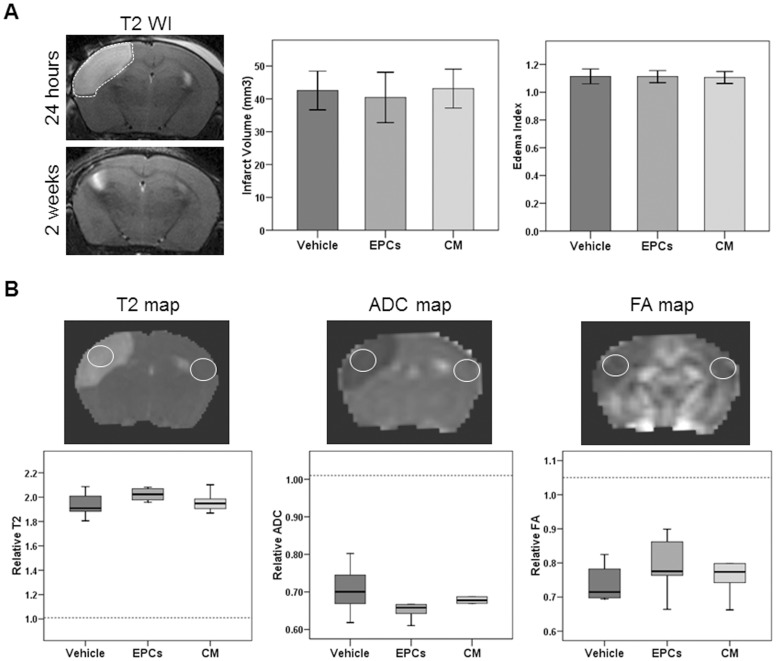
MRI after cerebral ischemia. Representative T2WI used to measure infarct volume at 24 hours (upper image, dashed lines in white) and representative T2WI of the brain at two weeks showing severe cortical damage; bar graphs represent infarct volume and edema index at 24 hours (**A**); data is presented as mean ± SD. Representative T2, ADC and FA maps of an ischemic animal and box-plots representing relative values (related to contralateral values) of ROIs; dashed line shows reference (median) for sham animals (**B**); data is expressed as median (IQR). Number of animals: n = 9 for vehicle, n = 9 for EPCs and n = 9 for CM and n = 3 for shams. No difference was found between ischemic groups.

A total of 38 animals were scanned 24 hours after ischemia (34 MCAO and 4 shams) before any treatment was given. Two mice died during MRI (related to uncontrolled body temperature) and four mice were excluded from the study for undersized infarcts (probably caused by electrocoagulation of only one of the branches of the distal portion of the MCA). Six animals included in the study were not scanned, because the MR scanner was not available at the last moment. A group of 15 mice were scanned two weeks after ischemia or sham surgery to monitor lesion extension and severity. However, since a complete necrosis and atrophy of the injured cortex was observed in ischemic mice (shown in Figure 1A) the following animals did not received a second scan.

Regions of interest (ROIs) of 1 mm^2^ were traced on T2, ADC and FA maps in all slices presenting infarcts (and inside the injured area). Measures of the corresponding contralateral hemisphere were also obtained. Finally, mean values were expressed as a ipsilateral/contralateral ratio, as described by others [17], [18].

### Treatment Groups

The neurorepair potential of different treatments was tested here in animals, to assess the possibility of giving them to stroke patients beyond the hyperacute phase. In order to avoid any potential confounding effect due to neuroprotection, treatments were administered one day after ischemia. Briefly, growing EPCs were washed twice at day 5–6 with EBM. Then, 4 mL of fresh EBM were added to obtain CM that was collected 24 hours later. CM was concentrated for 40 min using a 10 kDa filter unit (Millipore, Ireland), for a final volume of approximately 200 µL. At the same time, EPCs were trypsinized, counted and resuspended in 200 µL of EBM. Treatments were administered intravenously using a 30 G needle as follows: vehicle (n = 11, 200 µL of EBM), EPCs (n = 11, 200 µL of cells in EBM), CM (n = 12, 200 µL of CM). Sham animals received 200 µL of EBM (n = 4). The total number of EPCs administered ranged from 10^4^ to 2×10^5^, since different cell cultures yield different number of EPCs; this allowed us to investigate any association between the amount of administered cells, and angiogenesis or recovery. Treatments were administered randomly at 30–32 hours post-ischemia, after the first MRI session, and all animals received intraperitoneal injections of 5-bromo-2-deoxyuridine (BrdU; Sigma, MO USA, 50 mg/Kg), starting five days after ischemia and until sacrifice. One animal receiving CM treatment died a week after surgery.

The same surgical procedure was performed in a separate group of animals (n = 12) to analyze physiological variables. Treatments were administered intravenously 30 hours after ischemia as follows: vehicle (n = 3, 200 µL of EBM), EPCs (n = 3, 200 µL of cells in EBM), CM (n = 3, 200 µL of CM) and sham group (n = 3, 200 µL of EBM). Thirty minutes after treatment, cardiac arterial blood was drawn from the left ventricle and pH, blood gases (pO2 and pCO2), glucose, hemoglobin and electrolytes (Na^+^, K^+^ and HCO_3_^−^) were monitored using the i-STAT Portable Clinical Analyzer (Abbot). These animals all met the LDF criteria of arterial occlusion and reperfusion. Then, the brain was removed and the infarct volume measured using 2.5% of 2,3,5-triphenyl-2H-tetrazolium chloride staining as described previously [15], to confirm the extent of the infarct.

### Infarct Volume

Infarct volume and brain edema were determined using Image J. The hyperintense area corresponding to injured tissue and the complete areas of both ipsilateral and contralateral hemispheres were traced manually on T2WIs that were obtained one day after cerebral ischemia (16 slices/brain) by researchers who were blinded to treatment. The value for each slice was corrected for section thickness (0.55 mm). An edema index was determined as the sum of all areas of the ipsilateral hemisphere divided by the sum of all areas of the contralateral hemisphere. Lesion volume was calculated as the sum of the injured area across all slices and corrected for edema as proposed by our group and others [15], [19]. Results were given in mm^3^.

### Behavioral Testing

Several tests were used to assess stroke severity and functional outcome before MCAO (pre-MCAO), at 24 hours, at one week, and at two weeks. Initially, a modified neurological severity score was used to assess stroke severity and functional outcome [20]. However the test failed in demonstrating an impairment of function at 24 hours in most animals, which led us to use another test. Therefore, the corner test and the grip strength meter were used to evaluate more specific neurological functions as described elsewhere [21]. Briefly, the corner test (used to assess sensorimotor and postural asymmetries after MCAO) consisted of two boards (30 cm×20 cm×1 cm) attached at a 30° angle with a small opening between the boards to encourage entry in the corner. Ten trials were performed per animal and left and right turns were recorded. A laterality index was calculated as described previously [15], [21]. The grip strength meter measures the maximum strength exhibited by the mouse forelimbs [21]. Briefly, animals were suspended by the tail and approached to the grid. Once the mouse grasped the center of the grid with both forelimbs the animal was pulled backwards in the horizontal plane. The force applied to the grid was recorded as the peak tension and measured in grams. Six measures were acquired per time point and the mean force was obtained for statistical analysis. Researchers who were blinded to treatment group performed the functional tests.

### Immunohistochemistry

Two weeks after ischemia, mice were deeply anesthetized with isoflurane, the brains were transcardially perfused with 4% paraformaldehyde and fixed overnight at 4°C. Afterwards, brains were cryoprotected with 30% sucrose in PBS for 24 hours and frozen using dry ice and embedded in optimal cutting temperature (OCT), before storage at −80°C. Twelve µm-thick coronal sections (n = 9) were obtained serially, starting after the olfactory bulb, and every 400 µm. Immunostaining with antibodies for endothelial cells (Lectin; VectorLabs, USA or CD-31; Abcam, UK), proliferating cells (BrdU; Abcam, UK), mature neurons (NeuN; Millipore, IE) and neuroblasts (doublecortin, DCX; Abcam, UK) was performed as follows: sections were thawed at room temperature for 30 minutes and transferred to PBS for hydration. Then, cells were permeabilized with 0.3% triton X-100 for 10 minutes and blocking buffer (1% BSA and 5% goat serum) was applied for one hour. Primary and secondary antibodies were applied in blocking buffer and slices were finally mounted in Vectashield^®^ with DAPI to counterstain cell nuclei. BrdU-stained sections were pretreated with 2 M HCl for 30 min to open the DNA structure and neutralized with 0.1 M borate buffer (pH 8.4), before incubation in blocking buffer. Vessel density and neurons were measured in peri-infarct cortical areas adjacent to the damaged tissue using Image J. Briefly three slices separated by 1-mm distance were stained for Lectin or NeuN. Four images of the peri-infarct and contralateral cortex were captured with an inverted fluorescent microscope at 100× (Olympus, IX71). Finally, a total of 12 fields, including different areas, were used to compute the mean vessel density by a researcher who was blinded to treatment. Data were corrected by the signal of the corresponding contralateral hemisphere and expressed as a ratio. DCX and BrdU co-staining were only performed in sections including the subventricular zone (SVZ).

### Axonal Fiber Histology

Axonal fiber pathways were examined using Bielschowsky’s staining method. Briefly, slides were placed in 20% silver nitrate in the dark, and then ammonium hydroxide was added until the tissue turned brown with a yellow background. Slides were then washed in 95% ethanol, absolute ethanol and xylene, and then mounted in mounting medium. This stain shows axons in black. Positive stain was used to measure the thickness of the axon track emerging from the corpus callosum (CC) at the level of the infarct boundary by a neuropathologist who was blinded to treatment. The same measure was taken from the contralateral structure at the same level for data normalization. Thickness was expressed as a ratio between the ipsilateral and contralateral hemisphere measures.

### Multiplex ELISA

Additional EPC-secreted CM was obtained as described above through six independent EPC cultures. After concentration, CM was stored at −80°C until use. The content of EPC-secreted growth factors was determined using SearchLight® Angiogenesis Array 2 (Aushon Biosystems, MA, USA) for the simultaneous measurement of four angiogenic factors: PDGF-bb, HGF, FGF-b and VEGF. Cross-reaction with mouse proteins was confirmed for VEGF and PDGF-bb, whereas no data was available for HGF or FGF-b. Samples were assayed twice and the mean value of the two measurements was calculated. The mean intra-assay coefficients of variation were <20%. The images were analyzed with Array Analyst (Imaging Research, USA). The sensitivity limits were 1.0 pg/ml for PDGF-bb, 3.1 pg/ml for HGF, 2.0 pg/ml for FGF-b, and 4.9 pg/ml for VEGF. Importantly, concentrated EBM was also tested as a blank sample. Additionally, a Bradford assay was performed to determine total protein concentration and the values were normalized to pg of factor/µg of protein. Assays were performed by personnel blinded to treatment.

### Statistical Analysis

SPSS 15.0 was used for all statistical analyses. Shapiro-Wilk or Kolmogorov-Smirnov tests were used to verify the normal distribution of the variables. Statistical significance for intergroup differences for normally distributed variables was assessed using Student’s t-test or ANOVA followed by Fisher’s least significant difference (LSD) test. Non-normally distributed variables were analyzed using Kruskall-Wallis and Mann-Whitney tests. Repeated measure ANOVA was used to assess differences in functional recovery between time-points. Correlations were evaluated using Pearson’s coefficient for normally distributed variables. A p-value <0.05 was considered statistically significant at a 95% confidence level. Values from normally distributed variables were expressed as mean ± SD and represented as bar graphs, whereas values from non-normally-distributed variables were expressed as median (interquartile range) and represented as box-plots.

## Results

### Quantitative Characterization of MRI Parameters of Brain Injury

Infarct volume was similar in all groups at 24 hours of ischemia and before any treatment was administered (vehicle 42.5±5.8 mm^3^, EPCs 40.4±7.6 mm^3^ and CM 43.1±5.9 mm^3^) as shown in Fig. 1A. Sham animals had negligible lesions (0.6±1.04 mm^3^), probably due to the trauma of surgery. The edema index was also similar between ischemic groups at 24 hours (vehicle 1.1±0.05, EPCs 1.1±0.04 and CM 1.1±0.04; Fig. 1A), whereas the reference was 1±0.01 in sham animals. No difference was found in infarct volume or brain edema between ischemic groups, whereas all of them were significantly different when compared to the sham group (p<0.05). Severe lesions were observed at two weeks with a complete atrophy of the injured cortex (Fig. 1A, bottom image).

Measurements of T2, ADC and FA maps within the infarct core confirmed that the severity of the lesions was similar between all ischemic groups before any treatment was given (Fig. 1B). High T2 values were found to be similar between all ischemic groups: 1.90 (1.84–2.04) for vehicle, 2.02 (1.96–2.07) for EPCs and 1.94 (1.90–2.04) for CM, while the reference for sham animals was 1.01 (1–1.05). At the same time, low ADC values were detected in infarct areas of all ischemic groups: 0.70 (0.65–0.75) for vehicle, 0.65 (0.63–0.71) for EPCs and 0.67 (0.65–0.70) for CM, while the reference for sham animals was 1.01 (1–1.05). Finally, relative FA values were below the sham reference 1.05 (1.04–1.38) and similar between ischemic groups: 0.71 (0.69–0.80) for vehicle, 0.77 (0.73–0.86) for EPCs and 0.77 (0.70–0.86). No difference was seen among ischemic groups for any of the measured parameters at 24 hours and all of them were significantly different compared to the sham values (p<0.05), indicating similar ischemic lesions (Fig. 1B).

### Physiological Variables

The physiological variables measured in arterial blood 30 minutes after treatment administration remained unaltered among groups (see Table S1) and infarct volume was similar between these MCAO groups (vehicle 36.6±9.6 mm^3^, EPCs 35.8±13.9 mm^3^ and CM 41.1±5.9 mm^3^; p>0.05).

### Effects of EPC and Cell-Free Secreted Factors on Cortical Angiogenesis

**Figure 2:**
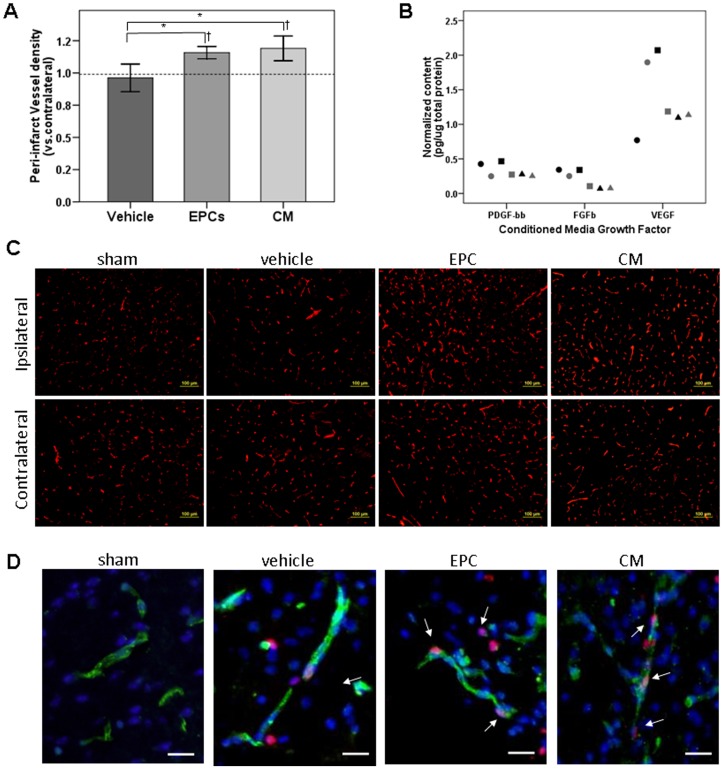
Peri-infarct angiogenesis after treatment. Bar graph representing vessel density (mean±SD vs. contralateral) in peri-infarct regions of ischemic animals treated with vehicle (n = 10), EPCs (n = 9) or CM (n = 6); dashed line shows reference (mean) for sham (n = 4) animals (**A**); * indicates p<0.05 vs. vehicle and † indicates p<0.05 vs. sham. Graph showing CM content of PDGF-bb, FGF-b and VEGF in six different cultures of EPCs represented as black or grey circles, squares or triangles (**B**). Representative micrographs of lectin immunofluorescence; bars represent 100 µm (**C**). Representative micrographs of CD-31 (green) and BrdU (red) staining merged with DAPI showing proliferating endothelial cells (arrows) in peri-infarct areas; bars represent 20 µm (**D**).

Angiogenesis was considerably enhanced in the cortical peri-infarct areas of animals receiving pro-angiogenic treatment. Our results show that vessel density was significantly increased in peri-infarct tissue at two weeks in EPC- and CM-treated animals, when compared to vehicle treatment (p<0.001 and p<0.0001, respectively) and to sham-operated animals (p = 0.003 and p = 0.001, respectively), as shown in Fig. 2A. Representative micrographs of peri-infarct lectin-positive vessels are shown in Fig. 2C. Double positive staining of CD-31 endothelial cells with BrdU was also seen in those peri-infarct areas as shown in Fig. 2D. Regarding the administered CM containing EPCs secretome, several growth factors were identified including VEGF (vascular endothelial growth factor) (1.36±0.5 pg/µg of total protein), FGF-b (fibroblast growth factor-basic) (0.2±0.13 pg/µg of total protein) and PDGF-bb (platelet-derived growth factor) (0.32±0.09 pg/µg of total protein), as shown in Fig. 2B. We were not able to detect HGF (hepatocyte growth factor) in the analyzed CM. Finally, no correlation could be found between vessel density and the number of administered EPCs or derived CM (r = 0.16 and r = −0.18, respectively; p>0.05).

### Axonal Reorganization and Neuroblast Migration

**Figure 3:**
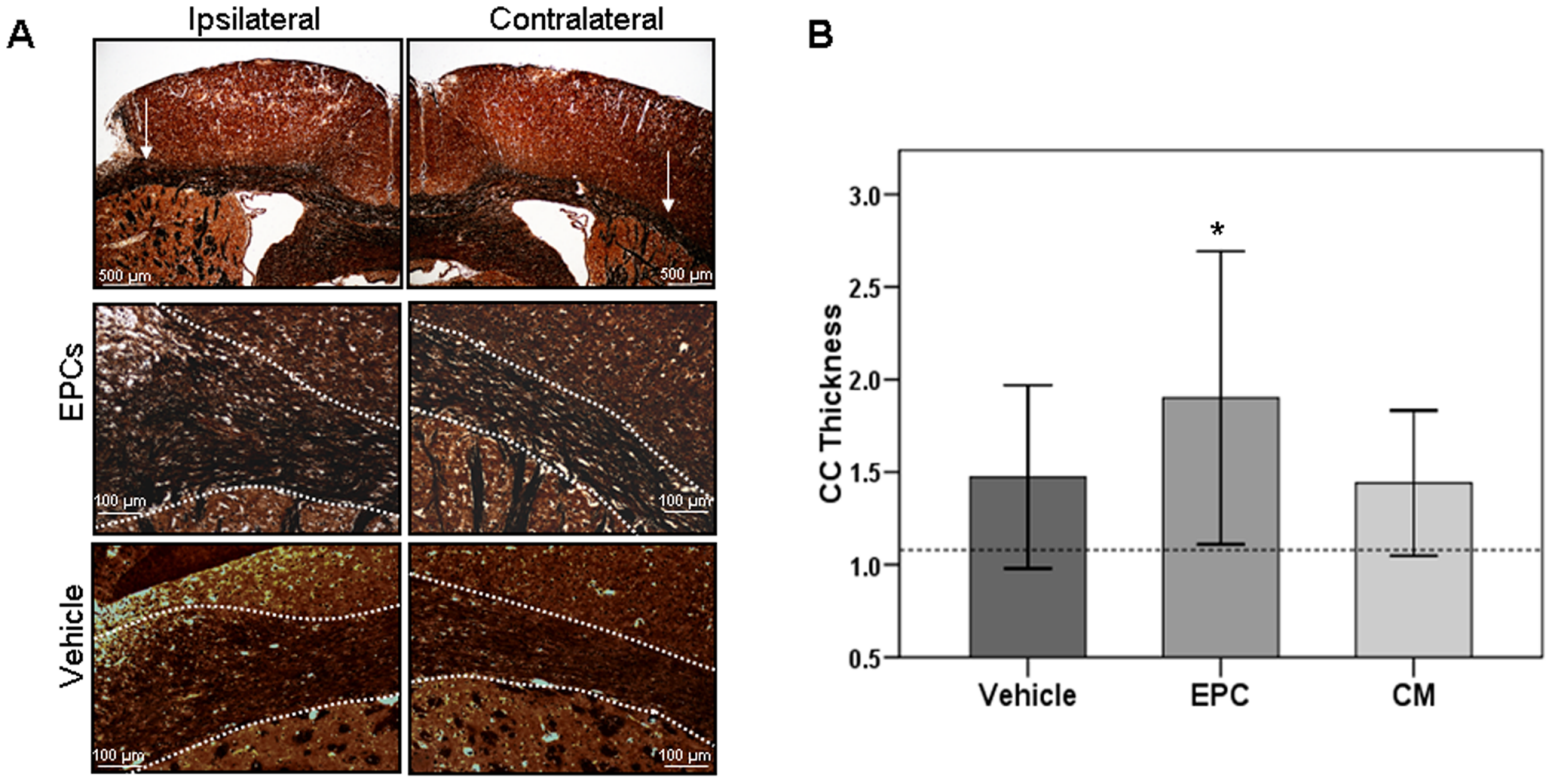
Representative images of Bielschowsky’s staining. Ipsilateral and contralateral hemispheres of vehicle and EPC-treated brains (**A**); bars represent 500 µm. White arrows in (**A**) indicate the region of the corpus callosum (CC) with increased thickness, with emerging axonal tracks shown in detail in the bottom panels. Bar graph representing the ipsilateral CC axonal thickness expressed as a ratio vs. the contralateral hemisphere (n = 10 for vehicle, n = 9 for EPCs and n = 7 for CM), dashed line shows reference (mean) for sham (n = 4) animals (**B**). Data is presented as mean±SD. * indicates p<0.05 vs. sham.

Histological examination revealed signs of axonal reorganization, as white matter tracks emerging from the corpus callosum were thicker at the level of the peri-infarct cortex in ischemic animals (Fig. 3A–B). The corpus callosum was thicker (with axons leaving the callosal track towards the peri-infarct) in animals that received EPCs than shams (1.90±0.79 vs. 1.08±0.22; p = 0.024), but not in vehicle-treated or CM-treated animals (Fig. 3B). No difference could be found when comparing ischemic animals (1.47±0.49 for vehicle, 1.90±0.79 for EPCs and 1.44±0.39 for CM; p>0.05). Moreover, no correlation was found between corpus callosum thickness and number of administered EPCs or derived-CM (r = 0.26 and r = −0.495, respectively; p>0.05) and no difference was detected between groups for contralateral measures (p>0.05).

**Figure 4:**
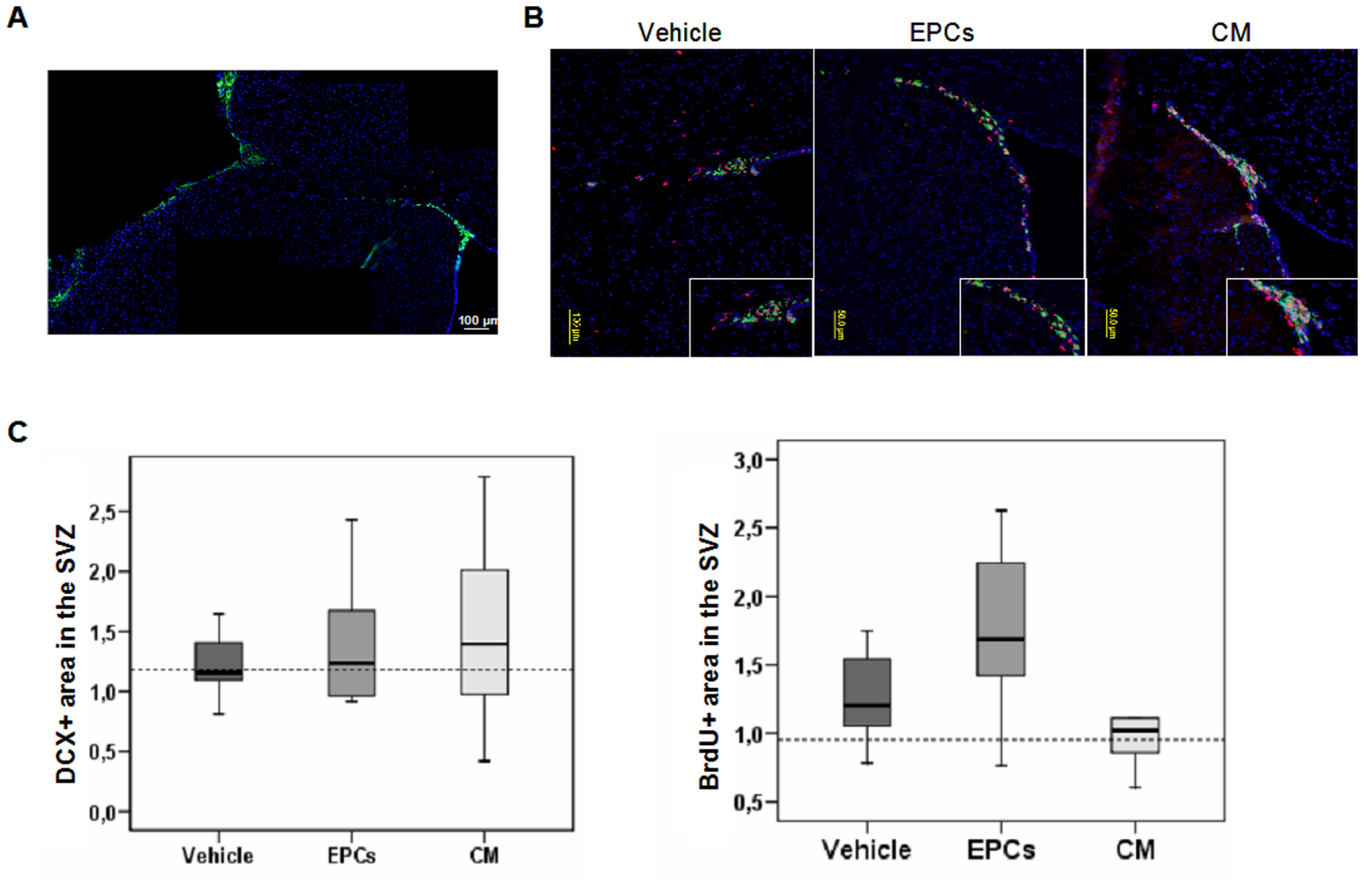
Immunofluorescence images showing neurogenesis at two weeks in the SVZ. Composition of four immunofluorescence images for DCX (green) and DAPI (blue) showing neuroblast migration from the SVZ towards cortical peri-infarct thorough the corpus callosum external capsule in an EPC-treated animal (**A**). Representative images of double immunofluorescence for DCX (green), BrdU (red) and DAPI (blue) are shown for the different treatment groups (**B**). Bottom right inserts show a magnification of the dorsolateral SVZ. Box-plots representing the percentage of DCX+ or BrdU+ area in the SVZ (n = 11 for vehicle, n = 11 for EPCs, n = 11 for CM and n = 3 for shams) (**C**); data is represented as median (IQR).

DCX-positive cells (a marker for neurogenesis) were identified in the dorsolateral SVZ zone of sham animals. However, in response to ischemia, the DCX-positive signal increased in migrating of neuroblasts towards peri-infarct regions; as shown in the image composition in Fig. 4A, which represents an EPC-treated animal. Migrating DCX-positive neuroblasts in the SVZ were particularly visible in brains from animals receiving CM treatment (Fig. 4B) and co-staining with BrdU indicated that most of these cells were also under proliferation (inserts in Fig. 4B). However the amount of neuroblasts expressed as DCX-positive area in the SVZ was not different between groups: 1.15 (1.08–1.56) for vehicle, 1.23 (0.95–1.68) for EPCs, 1.39 (0.93–2.13) for CM and 1.18 (0.66–1.51) in shams (p>0.05 as shown in Fig. 4C). The BrdU+ area in those SVZ were slightly increased in animals receiving EPCs (1.20 (1–1.55) for vehicle, 1.68 (1.19–2.35) for EPCs, 1.01 (0.77–1.34) for CM; p>0.05 as shown in Fig. 4C).

The presence of migrating neuroblasts was clearly seen in the border of the infarct area (Fig. 4A), but not in the contralateral cortex. In peri-infarct areas, DCX-positive cells were not under proliferation, as BrdU positive cells did not match with DCX signal. Cell counts for NeuN-positive mature neurons in peri-infarct areas did not reveal any significant difference among treatments (p>0.05).

### EPC-based Pro-angiogenic Therapy Improves Neurological Outcome

The grip strength meter test identified neurological deficits 24 hours after ischemia in all groups (n = 6/group) when compared to pre-MCAO values (post 82.4±13.2 vs. pre 111.1±17.2 for vehicle, p = 0.044; post 84.2±6.8 vs. pre 116.1±11.3 for EPCs, p = 0.002; post 83.5±8.7 vs. pre 105.3±8.2 for CM; P = 0.027).

Importantly, the forelimb strength of animals receiving EPCs or CM treatment was improved at one week and two weeks, as compared to the 24 h post-ischemia score (1 w 94.2±6.5 and 2 w 103.7±8.0 vs. post 84.2±6.8, p = 0.017 and p = 0.003 for EPCs; 1 w 92.8±10.1 and 2 w 91±10.9 vs. post 83.5±8.7, p = 0.049 and p = 0.026 for CM). Although spontaneous recovery could be observed in some vehicle-treated animals over time, this improvement was not significant for this group (1 w 99±16.3 and 2 w 97.4±17.7 vs. post 83.8±12.2; p = 0.226 and p = 0.302, respectively). The neurological score test failed to demonstrate any impairment in neurological function after ischemia in most animals, although large lesions could be observed by MRI. The corner test only showed neurological impairment one day after ischemia in the cohort of vehicle animals, when compared to pre-MCAO scores (p = 0.043). It failed to show impairment after ischemia in EPC- and in CM-treated groups (p = 0.157 and p = 0.273, respectively), although large lesions could be observed by MRI.

## Discussion

The present study confirms the angiogenic potential of EPC-secreted factors to safely treat cerebral ischemia beyond the hyperacute phase in a cell-free approach. Indeed, in a mouse model of stroke, we demonstrated that both EPCs and EPC-cell-free treatments significantly increased angiogenesis in peri-infarct areas where neurorepair should be potentiated. With regard to neurogenesis, our study could only demonstrate mild effects of the tested EPC-based treatments. Functional improvement at two weeks was also confirmed to be enhanced in mice receiving EPCs or CM. To the best of our knowledge, this is the first study to investigate the therapeutic potential of EPC-secreted factors in an *in vivo* model of cerebral ischemia.

In recent years, there has been growing interest in developing new neurorepair strategies for the treatment of stroke and other cardiovascular diseases. The narrow time window for neuroprotection, the identification of endogenous repair processes in the damaged brain, and the technical advances to obtain and grow many types of stem and progenitor cells have provided new opportunities for stroke treatment. In fact, phase I and II clinical trials using intracerebral transplantation of an immature neuron cell line [22], or intravenous transplantation of autologous mesenchymal stem cells [23] have already been conducted in stroke patients. Other studies have already confirmed that the intra-arterial administration of autologous bone marrow-MNCs days or months after stroke was also safe and feasible [24], [25]. However, to date, no study has proven the efficacy of these treatments on motor function or functional dependency in stroke patients.

Several studies have demonstrated the therapeutic potential of EPCs for the treatment of many ischemic diseases in experimental models. Regarding cerebral ischemia, Ohta et al. first demonstrated that the autologous intra-arterial transplantation of early bone marrow-derived EPCs after 90 minutes of ischemia reduced infarct volume and improved motor function [8]. In a mouse model of transient MCAO, Fan et al. showed again that the acute intravenous administration (at one hour) of human early EPCs was associated with a reduction in infarct volume and brain atrophy at day 3, a reduction in long-term neurological deficits, and an increase in vessel density [10]. More recently, it was reported for the first time that the administration of outgrowth populations of human EPCs improved neurological function in a rat model of ischemia-reperfusion. This improvement occurred along with an increase in angiogenesis, a decrease in apoptosis in peri-infarct areas and an increase in neurogenesis in the SVZ [9].

In accordance with these data, our results showed for the first time that the delayed administration of early EPCs (approximately 30 hours after permanent ischemia) could potentiate angio-vasculogenic responses in mice, considering that more vessels were formed in the peri-infarct areas two weeks after ischemia. More evidence of ongoing angiogenesis in the peri-infarct tissue is the presence of proliferating endothelial cells as shown by BrdU staining. Other authors have reported EPC-related angiogenesis occurring at this time point after cerebral ischemia in both rats and mice [9], [26]. No endogenous response could be observed in our Balb/c vehicle-treated animals in terms of angiogenesis after ischemia. This lack of response following a hypoxic stimulus has previously been described in Balb/c mice [27], and was explained by different strain responses following angiogenic triggers, or to a greater tolerance to hypoxia. Therefore, we believe that our capacity to enhance angiogenesis in Balb/c animals strengthens the importance of our proposed EPC-based therapies, since it is considered to be more difficult to stimulate angiogenic responses in this mouse strain.

This angiogenesis enhancement was even greater in animals receiving cell-free therapy with EPC-secreted factors. The exogenous administration of growth factors to potentiate angiogenesis has been proposed before to treat ischemic stroke. In fact, it has already been shown in animal models of stroke that the late (but not early) administration of growth factors such as VEGF enhanced angiogenesis in the ischemic brain by improving neurological recovery [28]. Also, HGF gene transfer reduced infarct volume and safely enhanced local angiogenesis, without disturbing the blood-brain barrier [29]. Our results showed that spleen-derived EPCs secrete important growth factors that improve angiogenesis, such as VEGF, FGF-b and PDGF-bb, and probably many others that have not been investigated in the present study, such as angiogenesis inhibitors. Other authors have also already postulated that a novel cell-free therapeutic approach for angiogenesis with *in vitro-*generated conditioned medium obtained from EPCs could be a potent alternative to progenitor cell therapies [30]. These authors demonstrated that an intramuscular injection of EPC-CM was as effective as cell transplantation for promoting tissue revascularization in a rat model of chronic hindlimb ischemia [30]. Similar results have been reported for the treatment of diabetic dermal wounds, were the injection of EPC-CM into wounded diabetic mice promoted wound healing and increased neovascularization to a similar extent to EPC transplantation [31]. In the present study, we demonstrated for the first time that angiogenesis was enhanced with the administration of EPC-CM in an animal model of stroke.

An important caveat in the cell transplantation field is that by administrating large amounts of cells (millions) systemically, preclinical studies have failed to clearly show massive stem/progenitor cell accumulation in brain vessels, or within the brain parenchyma. Usually, only isolated cells or small clusters are seen in the brain, despite the clear benefits demonstrated in terms of brain repair or neurological status. Our finding that treatment with EPC-secreted factors enhances angiogenesis as much as EPC treatment might explain the therapeutic effects of these cells, even if they do not anchor or infiltrate into the brain parenchyma. Importantly, and from a translational point of view, our group recently reported that human EPCs obtained from the peripheral blood of stroke patients secrete important growth factors including those identified in the present study [32]. Therefore the possibility to treat patients with autologous EPC-CM seems a promising therapeutic strategy to test in the near future.

In the present study, our aim was to demonstrate increased neurogenesis after EPC-based treatments, but only mild effects could be observed. The histological analysis of axonal tracks confirmed the enhancement of axonal reorganization towards cortical peri-infarct areas in animals receiving EPC cell therapy two weeks after the ischemic event. This enhancement occured near peri-infarct areas with enhanced angiogenesis and in close proximity with active areas of neuroblast migration. The coupling between angiogenesis and neurogenesis has been described to occur endogenously in peri-infarct areas after stroke [4], [33]. In fact, Ohab et al. elegantly demonstrated that these two processes are spatially linked in a neurovascular niche that is causally linked through the vascular production of growth factors such as stromal-derived factor-1 and angiopoietin-1 [4]. Our study demonstrated that EPC production of other growth factors, such as VEGF, FGF-b and PDGF-BB, could contribute to enhancing angiogenesis and neurogenesis in peri-infarct areas. The specific link between these two processes after EPC-based angiogenic therapy remains to be established.

Doublecortin is a 43–53 kDa microtubule binding protein required for the normal neural migration of immature neurons (neuroblasts) and is expressed in dividing neuronal precursor cells and immature neurons. Here, we noted only a mild increase in proliferating DCX-positive cells in the dorsolateral SVZ of animals treated with CM. However, we could not find a higher number of mature neurons (NeuN-positive) in animals receiving pro-angiogenic treatment, compared to vehicle-treated animals or shams. The spatial and temporal pattern of proliferation, migration and localization of neuroblasts in our study corresponded to what other authors have described to occur endogenously in animals with cortical infarcts [33]. However, further studies exploring longer time-points are needed to confirm whether or not the maturation into functional neurons occurs and the true effects of EPC-based therapies on neurogenesis.

Finally, endogenous axonal plasticity and functional recovery have been shown to be potentiated by the intracerebral transplantation of human neural progenitor cells one week after cerebral ischemia based on dendritic length, arborization, axonal sprouting and transport [34]. Moreover, the reparative effects of stem cell therapies seemed to be mediated by cell-secreted factors, such as VEGF [35]. In agreement with these data, our results support the importance of cell-free angiogenesis in stem/progenitor therapies, perhaps over the reparative potential of the cells *per se*. To our knowledge, our study is the first to report this reparative potential of EPC-secreted factors after stroke.

Our study had some limitations. First, the number of cells administered to the animals (and the derived CM) ranged from 10^4^ to 2×10^5^, because primary EPC cultures yielded different amounts of cells. However, this allowed us to establish that were was no association between the number of administered cells and vessel density or neuroblast extension. Therefore, increasing the number of cells should not increase the regenerative potential, at least for the tested range of cells. However, further studies are needed to investigate if larger numbers of cell could offer a better therapeutic approach. Second, our mice showed some spontaneous functional recovery, as seen in the vehicle group (although it did not reach statistical significance). We are aware that this point limits the power of the functional outcome recovery of our proposed pro-angiogenic treatments. In this regard, we want to highlight that to date, several tests have been demonstrated to be useful to assess short-term neurological deterioration in mice with cortical lesions. However, no robust data have been published for long-term deterioration [21]. Finally, we hypothesize that the mild effects found for neuronal remodeling and neurological recovery could be improved with other therapeutic approaches, such as by intracerebral administration of the proposed pro-angiogenic treatments. However, we believe that the translation of these neurorepair therapies for stroke to humans could be shorter if given intravenously, since this route of administration could present advantages in terms of safety and feasibility.

## Supporting Information

Table S1 Blood gas, electrolytes, glucose and hemoglobin measured 30 minutes after treatment during isoflurane anesthesia in 100% Oxygen.
(DOCX)

## References

[1] Molina CA (2011) Reperfusion therapies for acute ischemic stroke: current pharmacological and mechanical approaches. Stroke 42(1 Suppl): S16–19. 10.1161/STROKEAHA.110.598763 21164113

[2] Carmichael ST (2008) Themes and strategies for studying the biology of stroke recovery in the poststroke epoch. Stroke 39: 1380–1388. 18309162 10.1161/STROKEAHA.107.499962 PMC2711539

[3] Shen Q, Goderie SK, Jin L, Karanth N, Sun Y, et al (2004) Endothelial cells stimulate self-renewal and expand neurogenesis of neural stem cells. Science 304: 1338–1340. 15060285 10.1126/science.1095505

[4] Ohab JJ, Fleming S, Blesch A, Carmichael ST (2006) A neurovascular niche for neurogenesis after stroke. J Neurosci. 26: 13007–13016. 10.1523/JNEUROSCI.4323-06.2006 PMC6674957 17167090

[5] Pepper MS (1997) Manipulating angiogenesis. From basic science to the bedside. Arterioscler Thromb Vasc Biol 17: 605–619. 9108772 10.1161/01.atv.17.4.605

[6] Asahara T, Murohara T, Sullivan A, Silver, M; van der Zee R, et al (1997) Isolation of putative progenitor endothelial cells for angiogenesis. Science 275: 964–967. 9020076 10.1126/science.275.5302.964

[7] Taguchi A, Soma T, Tanaka H, Kanda T, Nishimura H, et al (2004) Administration of CD34+ cells after stroke enhances neurogenesis via angiogenesis in a mouse model. J Clin Invest 114: 330–338. 15286799 10.1172/JCI20622 PMC484977

[8] Ohta T, Kikuta K, Imamura H, Takagi Y, Nishimura M, et al (2006) Administration of ex vivo-expanded bone marrow-derived endothelial progenitor cells attenuates focal cerebral ischemia reperfusion injury in rats. Neurosurgery 59: 679–686. 16955050 10.1227/01.NEU.0000229058.08706.88

[9] Moubarik C, Guillet B, Youssef B, Codaccioni JL, Piercecchi MD, et al (2011) Transplanted Late Outgrowth Endothelial Progenitor Cells as Cell Therapy Product for Stroke. Stem Cell Rev 7: 208–220. 20526754 10.1007/s12015-010-9157-y

[10] Fan Y, Shen F, Frenzel T, Zhu W, Ye J, et al (2010) Endothelial progenitor cell transplantation improves long-term stroke outcome in mice. Ann Neurol 67: 488–497. 20437584 10.1002/ana.21919 PMC3026588

[11] Lodi D, Iannitti T, Palmieri B (2011) Stem cells in clinical practice: applications and warnings. J Exp Clin Cancer Res 30: 9. 21241480 10.1186/1756-9966-30-9 PMC3033847

[12] Seminatore C, Polentes J, Ellman D, Kozubenko N, Itier V, et al (2010) The postischemic environment differentially impacts teratoma or tumor formation after transplantation of human embryonic stem cell-derived neural progenitors. Stroke 41: 153–159. 19940279 10.1161/STROKEAHA.109.563015

[13] Yang Z, Di Santo S, Kalka C (2010) Current developments in the use of stem cell for therapeutic neovascularisation: is the future therapy “cell-free”? Swiss Med Wkly 140: w13130. 21170763 10.4414/smw.2010.13130

[14] Rosell A, Arai K, Lok J, He T, Guo S, et al (2009) Interleukin-1beta augments angiogenic responses of murine endothelial progenitor cells in vitro. J Cereb Blood Flow Metab 29: 933–943. 19240740 10.1038/jcbfm.2009.17 PMC3712840

[15] Morancho A, García-Bonilla L, Barceló V, Giralt D, Campos-Martorell M, et al (2012) A new method for focal transient cerebral ischemia by distal compression of the middle cerebral artery. Neuropathol Appl Neurobiol 38: 617–627. 22289071 10.1111/j.1365-2990.2012.01252.x

[16] Le Bihan D, Mangin JF, Poupon C, Clark CA, Pappata S, et al (2001) Diffusion tensor imaging: concepts and applications. J Magn Reson Imaging 13: 534–546. 11276097 10.1002/jmri.1076

[17] Jiang Q, Zhang ZG, Ding GL, Silver B, Zhang L, et al (2006) MRI detects white matter reorganization after neural progenitor cell treatment of stroke. Neuroimage 32: 1080–1089. 16860575 10.1016/j.neuroimage.2006.05.025

[18] Liu HS, Shen H, Harvey BK, Castillo P, Lu H, et al (2011) Post-treatment with amphetamine enhances reinnervation of the ipsilateral side cortex in stroke rats. Neuroimage 56: 280–289. 21349337 10.1016/j.neuroimage.2011.02.049 PMC3070415

[19] Yanamoto H, Hong SC, Soleau S, Kassell NF, Lee KS (1996) Mild postischemic hypothermia limits cerebral injury following transient focal ischemia in rat neocortex. Brain Res 718: 207–211. 8773789 10.1016/0006-8993(96)00122-9

[20] Guo Q, Wang G, Liu X, Namura S (2009) Effects of gemfibrozil on outcome after permanent middle cerebral artery occlusion in mice. Brain Res 1279: 121–130. 19427843 10.1016/j.brainres.2009.04.055 PMC2717616

[21] Rosell A, Agin V, Rahman M, Morancho A, Ali C, et al (2013) Distal occlusion of the middle cerebral artery in mice: Are we ready to assess long-term functional outcome? Transl Stroke Res 4: 297–307. 24323300 10.1007/s12975-012-0234-1

[22] Kondziolka D, Steinberg GK, Wechsler L, Meltzer CC, Elder E, et al (2005) Neurotransplantation for patients with subcortical motor stroke: a phase 2 randomized trial. J Neurosurg 103: 38–45. 16121971 10.3171/jns.2005.103.1.0038

[23] Bang OY, Lee JS, Lee PH, Lee G (2005) Autologous mesenchymal stem cell transplantation in stroke patients. Ann Neurol 57: 874–882. 15929052 10.1002/ana.20501

[24] Moniche F, Gonzalez A, Gonzalez-Marcos JR, Carmona M, Piñero P, et al (2012) Intra-arterial bone marrow mononuclear cells in ischemic stroke: a pilot clinical trial. Stroke 43: 2242–2244. 22764211 10.1161/STROKEAHA.112.659409

[25] Battistella V, de Freitas GR, da Fonseca LM, Mercante D, Gutfilen B, et al (2001) Safety of autologous bone marrow mononuclear cell transplantation in patients with nonacute ischemic stroke. Regen Med 6: 45–52. 10.2217/rme.10.97 21175286

[26] Hayakawa K, Pham LD, Katusic ZS, Arai K, Lo EH (2012) Astrocytic high-mobility group box 1 promotes endothelial progenitor cell-mediated neurovascular remodeling during stroke recovery. Proc Natl Acad Sci U S A 109: 7505–7510. 22529378 10.1073/pnas.1121146109 PMC3358881

[27] Ward NL, Moore E, Noon K, Spassil N, Keenan E, et al (2007) Cerebral angiogenic factors, angiogenesis, and physiological response to chronic hypoxia differ among four commonly used mouse strains. J Appl Physiol 102: 1927–1935. 17234796 10.1152/japplphysiol.00909.2006

[28] Zhang ZG, Zhang L, Jiang Q, Zhang R, Davies K, et al (2000) VEGF enhances angiogenesis and promotes blood-brain barrier leakage in the ischemic brain. J Clin Invest 106: 829–838. 11018070 10.1172/JCI9369 PMC517814

[29] Shimamura M, Sato N, Oshima K, Aoki M, Kurinami H, et al (2004) Novel therapeutic strategy to treat brain ischemia: overexpression of hepatocyte growth factor gene reduced ischemic injury without cerebral edema in rat model. Circulation 10: 424–431. 10.1161/01.CIR.0000109496.82683.49 14707023

[30] Di Santo S, Yang Z, Wyler von Ballmoos M, Voelzmann J, Diehm N, et al (2009) Novel cell-free strategy for therapeutic angiogenesis: in vitro generated conditioned medium can replace progenitor cell transplantation. PLoS One 4: e5643. 19479066 10.1371/journal.pone.0005643 PMC2682571

[31] Kim JY, Song SH, Kim KL, Ko JJ, Im JE, et al (2010) Human cord blood-derived endothelial progenitor cells and their conditioned media exhibit therapeutic equivalence for diabetic wound healing. Cell Transplant 19: 1635–1644. 20659357 10.3727/096368910X516637

[32] Navarro-Sobrino M, Rosell A, Hernández-Guillamon M, Penalba A, Ribó M, et al (2010) Mobilization, endothelial differentiation and functional capacity of endothelial progenitor cells after ischemic stroke. Microvasc Res 80: 317–323. 20594997 10.1016/j.mvr.2010.05.008

[33] Ohab JJ, Carmichael ST (2008) Poststroke neurogenesis: emerging principles of migration and localization of immature neurons. Neuroscientist 14: 369–380. 18024854 10.1177/1073858407309545

[34] Andres RH, Horie N, Slikker W, Keren-Gill H, Zhan K, et al (2011) Human neural stem cells enhance structural plasticity and axonal transport in the ischaemic brain. Brain 134: 1777–1789. 21616972 10.1093/brain/awr094 PMC3102243

[35] Horie N, Pereira MP, Niizuma K, Sun G, Keren-Gill H, et al (2011) Transplanted stem cell-secreted vascular endothelial growth factor effects poststroke recovery, inflammation, and vascular repair. Stem Cells 29: 274–285. 21732485 10.1002/stem.584 PMC3524414

