# Supplementary material for: Factors Secreted by Endothelial Progenitor Cells Enhance Neurorepair Responses after Cerebral Ischemia in Mice
Source: PLoS One. 2013 Sep 4;8(9):e73244. doi: 10.1371/journal.pone.0073244 (PMC3762828; doi:10.1371/journal.pone.0073244)
Supplement: Table S1 — Blood gas, electrolytes, glucose and hemoglobin measured 30 minutes after treatment during isoflurane anesthesia in 100% Oxygen. (DOCX) [file pone.0073244.s001.docx]

**Table S1. Blood gas, electrolytes, glucose and hemoglobin measured 30 minutes after treatment during isoflurane anesthesia in 100% Oxygen.**

| **Parameter** | **Vehicle** | **EPCs** | **CM** | **Sham** |
| --- | --- | --- | --- | --- |
| **pH** | 7.1 ± 0.0 | 7.1 ± 0.0 | 7.1 ± 0.0 | 7.1 ± 0.0 |
| **pCO_2_ (mmHg)** | 74.5 ± 1.5 | 71.7 ± 4.7 | 68.7 ± 6.3 | 78.1 ± 13.3 |
| **pO_2_ (mmHg)** | 96.6 ± 31.0 | 93.3 ± 49.0 | 106.0 ± 53.7 | 139.0 ± 24.6 |
| **Na^+^ (mmol/L)** | 148.6 ± 2.0 | 145.6 ± 1.5 | 147.0 ± 2.0 | 148.0 ± 1.0 |
| **K^+^ (mmol/L)** | 4.7 ± 0.4 | 4.5 ± 0.3 | 4.4 ± 0.3 | 4.7 ± 0.4 |
| **Glucose (mg/dL)** | 159.6 ± 3.7 | 188.0 ± 21.6 | 172.0 ± 33.6 | 196.0 ± 33.7 |
| **HCO_3_^-^ (mmol/L)** | 26.06 ± 0.2 | 23.8 ± 1.9 | 26.0 ± 2.0 | 26.3 ± 2.4 |
| **Hemoglobin (g/dL)** | 13.5 ± 0.7 | 13.8 ± 2.3 | 13.1 ± 0.5 | 13.8 ± 0.1 |

Note: Results are expressed as mean ± standard deviation. No significant differences were observed between treatments (n=3/group).
